# Preventive Orientation of Iraqi Dentists in Baghdad in 2016

**Published:** 2017-09

**Authors:** Fatin Abd Alsttar Aledhari, Katayoun Sargeran, Mahdia Gholami, Ahmad Reza Shamshiri

**Affiliations:** 1 MSc Student, Department of Community Oral Health, School of Dentistry, International Campus, Tehran University of Medical Sciences, Tehran, Iran; School of Dentistry, Anbar University, Anbar, Iraq; 2 Assistant Professor, Department of Community Oral Health, School of Dentistry, International Campus, Tehran University of Medical Sciences, Tehran, Iran; 3 Assistant Professor, Research Center for Caries Prevention, Dentistry Research Institute, Tehran University of Medical Sciences, Tehran, Iran; Department of Community Oral Health, School of Dentistry, Tehran University of Medical Sciences, Tehran, Iran

**Keywords:** Knowledge, Attitude, Dentists, Preventive Dentistry, Iraq

## Abstract

**Objectives:**

The aim of the present study was to assess the preventive orientation of Iraqi dentists in terms of their “knowledge” and “attitude” towards caries prevention and to explore their “preventive practice”.

**Materials and Methods:**

A cross-sectional study based on a self-administered questionnaire was conducted among 159 dentists who worked in Baghdad during the summer of 2016. The questionnaires obtained information on variables such as knowledge and attitude towards preventive dentistry, preventive practice and demographic variables and were distributed during the official working time. Logistic and multiple regressions served for statistical analyses.

**Results:**

From all the respondents, 71% were females and the response rate was 94% (n=150). The mean age was 40.75±9.88 years (range 27–65 years). After checking for completeness, 90 questionnaires remained for data analysis. The most positive attitude towards preventive dental care was related to the question: “whether preventive dentistry is essential for the community” (n=75, 83%). The highest knowledge was reported in response to three questions: “frequency vs. amount of sugar consumption”, “effect of sealant on caries prevention of newly erupted molars” and “effect of dental problems on general health” (n=83, 92.2%). The regression analysis showed a significant association between attendance in the continuing educational courses and preventive practice (P=0.03).

**Conclusions:**

To improve preventive dental orientation of Iraqi dentists, dental schools should put more emphasis on the topics about preventive dentistry. This will consequently improve their practice and oral health of the community.

## INTRODUCTION

The world still faces a huge burden of oral diseases especially in developing countries. This relates to many factors such as life-style, socio-economic status and poor public and professional knowledge about caries prevention and oral health promotion. Improved oral health requires up-to-date scientific evidence to control oral diseases [1,2].

In order to improve the public perception about prevention of oral diseases, the public should be informed about the causes of oral diseases especially dental caries [3]. The first step in improving the perception of the public is to train oral health professionals especially dentists, in this regard. Knowledge and attitudes attained by dentists during the academic dental education period about the importance of having preventive concepts, enables them to provide people with the best community and individual-based preventive activities [4,5]. Preventive dentistry is not a new topic, and has a history of more than 100 years; however, it has been more emphasized since the past century [6]. The preventive orientation of dentists themselves is one of the important factors through which they will be able to provide and practice preventive dental care. Several studies have shown that the knowledge of dentists about preventive dental care is not satisfactory [7,8]. The preventive dental care attitude and activities are lower among male and older dentists [9,10]. Dentists’ preventive orientation towards the public is determined by their practice, which is affected directly by their preventive attitude and knowledge [11]. Moreover, since dentists are symbolized as the source of oral health information in the community, their own knowledge is very important. Obviously, educational programs about caries prevention will enhance the attitude, knowledge and practice of dentists [12,13]. Beside exploring the preventive orientation of dentists, investigating their perceived barriers that can hinder preventive dental work, may provide solutions to overcome these obstacles [14,15]. If preventive strategies are properly implemented in the health care system of Iraq, dental workforce especially dentists, have to be well oriented on preventive dental care. Some epidemiological studies were conducted in Iraq concerning dental caries and its related factors; however, most of them dealt with preschool or primary school children [16–18]. The DMFT index for the age group of 12-year-olds in Iraq ranges from 1.2 to 2.5 [19,20].

Public oral health services are provided in specialty and primary health care (PHC) centers in Baghdad, and are completely free of charge. All kinds of dental services, including general and special care, are provided in specialty centers, while only primary care is provided in PHC centers. General dentists recently graduated from dental schools must work in specialty centers under supervision for one year. Almost all the dentists in Baghdad work in public centers and a small percentage have private practice as well. No study has been previously done about the preventive orientation of Iraqi dentists. Lack of accurate information necessities continuous research in this area, which will help us know the best approach towards preventive strategies for improving community dental services for the oral health improvement of Iraqi population. The aim of the present study was to investigate the preventive orientation of Iraqi dentists in Baghdad in terms of their “knowledge” and “attitude” towards caries prevention and to explore their “preventive practice”.

## MATERIALS AND METHODS

### Ethical considerations

The study was conducted on a voluntary basis. Dentists, who gave their verbal consent, participated in the study, after obtaining all the agreements from the Iraqi Ministry of Health in Baghdad. The study followed World Medical Association Declaration of Helsinki - Ethical Principles for Medical Research Involving Human Subjects. Ethical approval was obtained from Tehran University of Medical Sciences Research Ethics Committee (ID number: IR.TUMS.VCR.REC.1396.1984).

### Study design and population:

A cross-sectional study based on a self-administered questionnaire was conducted among Iraqi dentists in Baghdad. The study population comprised of general dental practitioners (GDPs) who worked in Specialty Dental Centers (n=18) and PHC centers in Baghdad. A convenient sampling method was adopted to select the GDPs from the centers. Security concerns resulted in convenient sample selection from secure areas of Baghdad city. We excluded preventive and prosthetic dental centers because of the nature of their activities, and two other centers because of the security issues in Baghdad; therefore, in order to complete our sample size we included GDPs who worked in PHC. The response rate was 94% (n=150) from 159 questionnaires distributed.

However, only 90 questionnaires that contained complete data were included in the analyses (completeness 60%).

### Sample size:

The following formula was used to calculate the sample size for estimating knowledge or attitude in the study population. According to the study by Ghasemi et al [10], we put the standard deviation (SD) equal to 1.93 (z= 1.96, α= 0.05, d = 0.3).

$$n={\left[\frac{Z_{1-\alpha /2}\times \mathrm{SD}}{d}\right]}^{2}={\left[\frac{1.96\times 1.93}{0.3}\right]}^{2}\approx 159$$

### Questionnaire and variables:

We used a valid and reliable questionnaire that was previously developed by Ghasemi et al [10]. The questionnaire consists of general information on variables such as age, gender, dental school, working in private sector, duration of practice as GDP, assistant type, history of previous attendance to continuing education programs, knowledge of and attitude towards preventive dental care and dentists’ self-reported preventive practices. Nine questions were used to measure dentists’ knowledge (Likert scale from 1 to 5). To measure dentists’ attitude towards prevention, semantic deferential method was used (Likert scale 1 to 7) [10]. Dentists’ preventive practices were assessed using “high risk (HR)” and “low risk (LR)” patient scenarios. The HR case, was defined as an 18-year-old boy with irregular tooth brushing, visible plaque, several cavities, and previous restorations. The LR case was a 22-year-old female medical student who brushed her teeth regularly and had only one restoration and one radiographically proven caries [10]. The participants were requested to specify their level of agreement about utilizing preventive actions for HR and LR [10].

### Statistical analysis:

The data had some missing values; the pattern was “missing at random” (missing in age was conditional to the gender).

Thus, STATA software was used to fill-in missing data using ‘impute’ command. The acceptance level of missing was ≤ 20% for each question. Independent variables such as age, dental school, working in private sector and attendance to the continuing education course about aries prevention were dichotomized. Questionnaire scores (knowledge, attitude and preventive practice) were standardized between 0 to 100 and then categorized to 3 categories of high (66.6–100), medium (33.3–66.6) and low (0–33.3); for attitude and preventive practice after categorizing to high, medium and low, we combined the two categories of low and medium and consequently had two categories: low-medium and high. Simple logistic regression was used to assess the association between the dependent variable (preventive practice) and independent variables of age, gender, dental school, duration of practice as GDP, educational course attendance, assistant type, attitude and knowledge (level of significance was set at P <0.20) [21]. Multiple logistic regression was used to assess potential predictors. Level of significance was set at P <0.05.

## RESULTS

From 159 questionnaires that were distributed, 150 dentists responded to the questions (response rate 94%). However, only 90 questionnaires that contained complete data were included in the analyses, of which 71.1% (n=64) had been answered by females (Table 1).

**Table 1.** Demographic characteristics of the participants (GDPs in Baghdad) (n=150/90)

	**Total GDPs n (%)**	**GDPs analyzed n (%)**
Gender		
Male	43 (28.7)	26 (28.9)
Female	107 (71.3)	64 (71.1)
Age		
≤40 yrs.	77 (51.3)	46 (51.1)
>40 yrs.	73 (48.7)	44 (48.9 )
Dental school from which the dentist was graduated from		
Baghdad	19 (12.7)	14 (15.6)
Others	131 (87.3)	76 (84.4)
Duration of practice as GDP		
<10 years	54 (36.0)	34 (37.8)
10–19 years	52 (34.7)	28 (31.1)
≥20 years	44 (29.3)	28 (31.1)
Private practice		
No	84 (56.0)	51 (56.7)
Yes	66 (44.0)	39 (43.3)
Assistant type		
Dental hygienist and dental nurse with university certificate	41 (27.3)	25 (27.8)
Assistant without university certificate	29 (19.3)	19 (21.1)
Secretary as assistant or no one	80 (53.3)	46 (51.1)
Attending continuing education courses		
No	34 (22.7)	21 (23.3)
Yes	116 (77.3)	69 (76.7)

### Knowledge:

The distribution of dentists’ knowledge in the three score categories was as follows: high (n=55, 38.5%), medium (n=84, 58.7%) and low (n=4, 2.8%). The majority of study participants agreed with the following statements: “dental problem can lead to general health problems”, “sealants are effective in prevention of pit and fissure caries in newly erupted molars” and “fluoridation of drinking water in regions with low fluoride is an effective, safe, and efficient way to prevent dental caries” (with the same agreement level; n=83, 92.2%). They showed the least knowledge regarding the statement that: “the use of fluoride toothpaste is more important than the brushing technique in caries prevention” (n=22, 24.7), “clinical examination of a newly erupted tooth with a sharp explorer will damage enamel rods and predispose the tooth to caries” (n=31, 34.4%) and also “a restored tooth is more likely to be lost than a sound one” (30.3%).

### Attitude:

The distribution of dentists’ attitude in the three score categories was as follows: high (n=67, 58.8%), medium (n=44, 38.6%) and low (n=3, 2.6%). The dentists revealed higher positive attitude towards preventive dental care in their perception as the preventive dentistry is useful for the community (n=75, 83.3%), essential for the community (n=71, 78.9%), and easy for dentists (n=60, 67.4%). The least positive attitude was regarding the perception that preventive dentistry is somewhat not attractive for dentists (n=37, 42.5%).

### Preventive practice:

Concerning preventive practice of the participants for HR scenario, most of the dentists who acquired high scores, were those who had been graduated from dental schools other than Baghdad (11/14; 78.6%, vs. 41/76; 53.9%) and those who had working experience as GDPs for less than 10 years (22/34; 64.7% vs. 15/28; 53.6%) (Table 2). Multiple regression analysis showed a significant association between attendance to the continuing education course (P=0.03) with preventive practice. Besides, an association was found between preventive practice and the dental school, in which the P value was approaching significance (P=0.07).

**Table 2.** Distribution (n, %) of participants (GDPs in Baghdad) (n=90) belonging to the categories of preventive practice for high risk patients and simple logistic regression results

	**Preventive practice (score)**		**OR**	**95% CI**	**P-value**

	**Low-Med**	**High**			
Gender					
Female	26 (40.6%)	38 (59.4%)	1.25	0.50–3.14	0.63
Male	12(46.2%)	14(53.8%)	Ref.		
Age (years)					
≤40	18 (339.1%)	28 (60.9%)	1.27	0.56–2.99	0.54
>40	20 (45.5%)	24 (54.5%)	Ref		
Dental school					
Baghdad	35 (46.1%)	41 (53.9%)	0.32	0.08–1.24	0.09
Others	3 (21.4%)	11 (78.6%)	Ref		
Duration of practice					
<10 years	12 (35.3%)	22 (64.7%)	Ref		
10–19 years	13 (46.4%)	15 (53.6%)	0.63	0.23–1.75	0.38
≥20 years	13 (46.4%)	15 (53.6%)	0.63	0.23–.75	0.38
Working in private sector					
No	23 (45.1%)	28(54.9%)	Ref		
Yes	15 (38.5%)	24 (61.5%)	1.31	0.56–3.07	0.53
Dental assistant					
Dental hygienist and dental nurses with university certificate	10 (40.0%)	15 (60.0%)	1.26	0.47–3.39	0.65
Assistant without university certificate	7 (36.8%)	12 (63.2%)	1.44	0.48–4.32	0.52
Secretary as assistant or no one	21(45.7%)	25 (54.3%)	Ref		
Education course attendance					
No	13 (61.9%)	8 (38.1%)	Ref.		
Yes	25 (36.2%)	44 (63.8%)	2.86	1.04–7.84	0.041
Attitude score					
Low and medium	20 (46.5%)	23 (53.5%)	Ref.		
High	18 (38.3%)	29 (61.7%)	1.140	0.61–3.24	0.43
Knowledge score					
Low	13 (50.0%)	13 (50.0%)	Ref		
Medium	20 (38.5%)	32 (61.5%)	1.60	0.62–4.14	0.33
High	5 (41.7%)	7 (58.3%)	1.40	0.35–5.57	0.63

For LR scenario, most of the younger dentists who acquired high scores, were those who aged 40 or less (39/46; 84.8%, vs. 32/44; 72.7%) and those with medium and high knowledge scores (44/52; 84.6% and 10/12; 83.3%, vs. 17/26; 65.4 %) (Table 3). Multiple regression analysis showed a significant association between level of knowledge in relation to positive preventive action (P=0.02) while the age revealed a marginally significant association (P=0.05).

**Table 3.** Distribution (n, %) of the participants (GDPs in Baghdad) (n=90) belonging to the categories of preventive practice for low risk patients and simple logistic regression results

	**Preventive Practice LR2**		**OR**	**95%CI**	**P-value**

	**Low-Med**	**High**			
Gender					
Female	12 (18.8%)	52 (81.3%)	1.59	0.55–4.66	0.39
Male	7 (26.9%)	19 (73.1%)	Ref.		
Age (years)					
≤40	7 (15.2%)	39 (84.8%)	2.09	0.74–5.93	0.17
>40	12 (27.3%)	32 (72.7%)	Ref		
Dental school					
Baghdad	17 (22.4%)	59 (77.6%)	0.58	0.12–2.84	0.50
Others	2 (14.3%)	12 (85.7%)	Ref		
Working duration					
<10 years	5 (14.7%)	29 (85.3%)	Ref		
10–19 years	6 (21.4%)	22 (78.6%)	0.63	0.17–2.34	0.49
≥20 years	8 (28.6%)	20 (71.4%)	0.43	0.12–1.51	0.19
Working in private sector					
No	12 (23.5%)	39 (76.5%)	Ref		
Yes	7 (17.9%)	32 (82.1%)	1.41	0.49–3.99	0.52
Dental assistant					
Dental hygienist and dental nurses with university certificate	5 (20.0%)	20 (80.0%)	1.11	0.33–3.71	0.86
Assistant without university certificate	4 (21.1%)	15 (78.9%)	1.04	0.28–3.85	0.95
Secretary as assistant or no one	10 (21.7%)	36 (78.3%)	Ref		
Education course attendance					
No	5 (23.8%)	16 (76.2%)	Ref.		
Yes	14 (20.3%)	55 (79.7%)	1.23	0.38–3.92	0.73
Attitude score					
Low and medium	10 (23.3%)	33 (76.7%)	Ref.		
High	9 (19.1%)	38 (80.9%)	1.28	0.46–3.53	0.63
Knowledge score					
Low	9 (34.6%)	17 (65.4%)	Ref		
Medium	8 (15.4%)	44 (84.6%)	2.91	0.97–8.79	0.05
High	2 (16.7%)	10 (83.3%)	2.65	0.47–14.78	0.27

## DISCUSSION

The aim of the present study was to investigate the preventive orientation of Iraqi dentists in Baghdad in terms of their “knowledge” and “attitude” towards caries prevention and to explore their “preventive practice”. The main results of the study can be summarized as follows.

The majority of the GDPs were aware that dental problems can lead to general health problems and also knew about the effectiveness of water fluoridation for prevention of dental caries. Most of the participants had positive attitude towards the concept that preventive dentistry is useful for the community. Knowledge and attending the continuing education courses on caries prevention were the most important factors affecting the preventive orientation of Iraqi GDPs.

The present study was the first to assess Iraqi dentists’ preventive orientation. As in other studies, we also had some limitations. The first and the most important was security concerns which resulted in convenient sample selection from secure areas of Baghdad city. However, only two dental centers were excluded from the study for this reason. Another limitation was time; since the survey was conducted during the official working hours for dentists. Consequently, most dentists were reluctant to participate in the study or fill the questionnaire completely.

In general, the participants of the present study did not have adequate knowledge and attitude regarding preventive dental care. This was in line with the findings of several studies [7–8,22–24]. According to the study by Wagle et al, [7] most Nepali dentists had good knowledge about the importance of fluoride use for dental caries prevention, which is similar to the findings of our study. Nowadays, dentists have an ideal opportunity to provide evidence-based preventive practice. Their knowledge, attitude and perceived values about prevention will aid them in clinical practice. Knowledge about prevention is associated with good preventive attitude and practice, which are not always seen in dentists. This may be due to missing evidence-based guidelines in applying preventive measures [25]. According to the findings of this study, male and female dentists were not significantly different in terms of knowledge and attitude, which was in line with the findings of the study conducted by Ghasemi et al. [10] regarding Iranian dentists. In the study by Yusuf et al, [9] they found no significant association between gender and attitude; although females had a more positive attitude. This was in line with the findings of our study.

The study results demonstrated that dentists who attended continuing education courses and those who had higher knowledge of preventive dental care, reported better preventive practice. Most of the dental schools today spend more time in teaching the recent evidence on prevention [26]. Insufficient preventive education can be one of the barriers for the preventive practice of dentists and in providing preventive practices in the community [27]. Dentists should acquire this knowledge from the early years of their study and this knowledge should be developed in their professional life, by continuing education courses on caries prevention [28].

The study results showed that Iraqi dentists who graduated from dental schools/universities other than the University of Baghdad and those who were younger, reported better preventive practice. This will be of value to investigate the causes in order to include proper educational courses and revise dental curriculum where needed for undergraduate dental students and to design special continuing education courses focused on preventive dentistry.

Although the acquired results are not generalizable to the whole Iraqi dentists’ population, they provide an insight to their preventive practice. Identifying the determinants of preventive orientation of dentists can be a subject of future research. We recommend establishment of a surveillance system for the assessment of GDPs’ preventive practice in Iraq, in order to evaluate their needs and to empower them in providing high quality scientific preventive dental care for the population.

## CONCLUSION

To improve preventive dental orientation of Iraqi dentists, dental schools should put more emphasis on the topics about preventive dentistry in general dental education. Continuing education courses will help to update evidence-based knowledge and practice of dentists. This will consequently improve the oral health of the community.
